# Genetically Epilepsy-Prone Rats Display Anxiety-Like Behaviors and Neuropsychiatric Comorbidities of Epilepsy

**DOI:** 10.3389/fneur.2018.00476

**Published:** 2018-06-27

**Authors:** Brittany L. Aguilar, Ludise Malkova, Prosper N'Gouemo, Patrick A. Forcelli

**Affiliations:** ^1^Interdisciplinary Program in Neuroscience, Georgetown University, Washington, DC, United States; ^2^Department of Pharmacology and Physiology, Georgetown University, Washington, DC, United States; ^3^Department of Pediatrics, Georgetown University, Washington, DC, United States; ^4^Department of Neuroscience, Georgetown University, Washington, DC, United States

**Keywords:** anxiety, comorbidity, seizures, audiogenic seizures, depression, rat models

## Abstract

Epilepsy is associated with a variety of neuropsychiatric comorbidities, including both anxiety and depression. Despite high occurrences of depression and anxiety seen in human epilepsy populations, little is known about the etiology of these comorbidities. Experimental models of epilepsy provide a platform to disentangle the contribution of acute seizures, genetic predisposition, and underlying circuit pathologies to anxious and depressive phenotypes. Most studies to date have focused on comorbidities in acquired epilepsies; genetic models, however, allow for the assessment of affective phenotypes that occur prior to onset of recurrent seizures. Here, we tested male and female genetically epilepsy-prone rats (GEPR-3s) and Sprague-Dawley controls in a battery of tests sensitive to anxiety-like and depressive-like phenotypes. GEPR-3s showed increased anxiety-like behavior in the open field test, elevated plus maze, light-dark transition test, and looming threat test. Moreover, GEPR-3s showed impaired prepulse inhibition of the acoustic startle reflex, decreased sucrose preference index, and impaired novel object recognition memory. We also characterized defense behaviors in response to stimulation thresholds of deep and intermediate layers of the superior colliculus (DLSC), but found no difference between strains. In sum, GEPR-3s showed inherited anxiety, an effect that did not differ significantly between sexes. The anxiety phenotype in adult GEPR-3s suggests strong genetic influences that may underlie both the seizure disorder and the comorbidities seen in epilepsy.

## Introduction

Epilepsy is associated with a variety of neuropsychiatric comorbidities, including both anxiety and depression (1, 2). Despite the presence of both comorbidities in epilepsy, most studies have focused solely on the depression phenotype, resulting in reference to anxiety as the “forgotten psychiatric comorbidity” (3). In fact, persons with epilepsy have an approximately two-fold increase in prevalence of generalized anxiety disorder (2), which may contribute to reduced quality of life (4). This underscores the need for further examination of anxiety in epilepsy and accentuates the importance of investigating common pathophysiology of both anxiety and epilepsy.

The etiology of anxiety in epilepsy remains unresolved. We suggest that at least two hypotheses can explain the high rate of comorbidity, (1) the pathology and circuit disruptions that lead to seizures also lead to the emergence of anxiety, and (2) recurrent seizures lead to emergence or exacerbation of anxiety [for further discussion of the “chicken or egg” problem with respect to epilepsy and comorbidities, see (5)]. In the clinic, the contributions of these factors are difficult to dissociate, necessitating preclinical models in which these features are separable. Here, rodent models of epilepsy provide a method for this assessment. In several strains of rats with inherited epilepsies, behavioral comorbidities (most notably depressive-like symptoms) have been reported; these include the genetic absence epilepsy rats from Strasbourg (GAERS) (6) and Wistar Albino Glaxo/Rijswijk (WAG/Rij) rats (7), both strains that display spontaneous absence seizures. However, in these studies, behavior was assessed after the onset of spontaneous seizures (i.e., on the background of repeated seizure history), making it impossible to dissociate the contribution of ongoing seizure activity and that of underlying genetics. Thus, while these data support the hypothesis that seizure activity *per se* can modulate the expression of anxiety-like and depression-like behaviors, they do not directly address the role of underlying pathology or genetic predisposition.

Acoustically evoked seizure (or audiogenic seizure, AGS) susceptible strains offer an opportunity to evaluate the contribution of genetic predisposition of seizures. In these models, behaviors can be assessed in animals that have no or minimal seizure history (8). One strain of interest is the genetically epilepsy-prone rat (GEPR-3) that exhibits inherited susceptibility to tonic-clonic seizures. The seizure susceptibility in GEPR-3s is associated with a deficit in both noradrenergic and serotonergic signaling, a profile similar to humans with depression (9, 10). Moreover, we have recently reported volumetric alterations in midbrain networks associated with defense behavior and anxiety in GEPR-3s (11). Finally, casual observations across independent laboratories using the GEPR-3s have reported increased aggression and anxiety-like responses to human handling. However, no experimental data have been published to confirm anxiety or depression-like comorbidities in this strain.

To address these gaps in knowledge, we evaluated the profile of anxiety- and depression-like behaviors in female and male adult GEPR-3s, as compared to female and male Sprague-Dawley (SD) rats. Despite the prevalence of sex-specific differences in neuropsychiatric disorders and the relationship they may have to incidence of epilepsy (3, 12, 13), sex has not been evaluated as a factor in prior studies of epilepsy comorbidity in animal models.

## Materials and methods

### Animals

Three-month-old male and female SD rats and GEPR-3s were used (10–12 per group). The same animals were tested on a within-subject basis on the tasks described below. SD rats were obtained from Harlan Labs and GEPR-3s were obtained from our animal colony maintained at Georgetown University. All animals were housed in a temperature/ humidity-controlled room on a 12 h/12 h light/dark cycle with free access to food and water. All efforts were made to minimize the number of animals used in these experiments. This study was carried out in accordance with the recommendations of the NIH Guide for the Care and Use of Laboratory Animals. The protocol was approved by the Georgetown University Animal Care and Use Committee.

### Confirmation of audiogenic susceptibility

To ensure penetrance of AGS phenotype, GEPR-3s were tested once for seizures at postnatal day 21 (PND 21). GEPR-3s are exposed to an acoustic stimulus (100–110 decibels, sound pressure levels pure tones) that was presented until seizure was elicited (or 60 s if no seizure was observed) (14). All GEPR-3s exhibited wild running that evolved into bouncing tonic-clonic seizures. Although GEPR-3s have been exposed to one AGS episode as a required screening test for inherited seizure susceptibility, they are naïve to repetitive AGS. It is unlikely that a single AGS episode at PND 21 can account for all subsequent comorbidity phenotypes seen at PND 90, however, we acknowledge the limitation of this methodology. In future studies, examining seizure susceptibility *after* the completion of behavioral testing would allow address this possible concern.

### Behavioral testing

All behavioral tests were performed consecutively, on a within-subject basis, in the order described below. Twenty to forty eight hours elapsed between two tests. Prior to each test day, animals were transported from the vivarium to the testing room. Animals were allowed a minimum of 30 min to acclimate to the testing environment prior to initiating testing. The test apparatuses were sanitized (with 70% ethanol solution) between animals. Behavioral tests were conducted in the order specified below, i.e., Open Field test, Elevated Plus Maze (EPM), Light-Dark Transition test, Looming Threat test, Acoustic Startle response, Sucrose Preference test, Novel Object Recognition test, Electrical stimulation of DLSC. Ten to 15 days after the end of behavioral tests, male GEPR-3s (*n* = 10) and SD rats (*n* = 10) were implanted with a single electrode targeted to medial deep layers of the superior colliculus (DLSC) to evaluate defense responses caused by midbrain activation (see below for details).

### Open field test

Open field testing was performed to measure spontaneous activity in rodents (15, 16). In this test, the desire to explore the novel arena is pitted against the species-typical response to avoid open spaces. Animals were placed into a Plexiglass enclosure (16′′ × 16′′ × 16′′, TruScan Arena, Coulbourn Instruments, Whitehall, PA) with 775 lux illumination over the center of the arena. A square (8.5′′ × 8.5′′) was drawn in the floor of the arena, forming the “inner” portion of the open field. Animals were allowed to explore for 10 min, during which total distance traveled and inner/outer entries were recorded using ANYmaze software (Stoelting Co., Wood Dale, IL), as previously described (17). Data for one animal (male, SD rat) were not recorded by the tracking software, thus this animal was excluded from open field analysis and subsequent correlation analyses.

### Elevated plus maze

Elevated plus maze (EPM) testing was performed and scored as previously described (18, 19) using a standard gray rat EPM (50 cm arms, elevated 40 cm off the ground (Stoelting Co., Wood Dale, IL). This test pits the desire to explore the novel maze against the species-typical preference to avoid open, elevated spaces as compared to enclosed spaces, and has been established as a tool for assessment of anxiety in rats (20, 21). Testing was conducted under 20 lux red light. The test lasted 300 s. The number of arm entries, time spent in open and closed arms, head pokes from the closed to open arms, head dips off the maze from the open arms, and stretch-attend postures were recorded using ANYmaze software (Stoelting Co, Wood Dale, IL). Based on previous studies in epilepsy models, head pokes and dips were used as an ethological parameter (22, 23). Stretch-attend posture was categorized as either “protected” when occurring when the body was positioned in the closed arms, or “unprotected” when the body was positioned in the open arms.

### Light-dark transition test

Light-dark transition testing was conducted as we have previously described (24, 25). As with the EPM, this test pits rats' innate aversion to bright areas against their natural drive to explore in response to mild stressors such as a novel environment. While originally described as a test of anxiolytic sensitivity in mice, this task has also been validated in rats (26, 27). Animals were placed into a testing apparatus (San Diego Instruments) that was half open and half covered by a black box with an opening for animals to enter. Ambient illumination of the room was 775 lux. Animals are initially placed in the light side of the apparatus facing the door to the “dark” chamber and filmed for 5 min. Latency to initially cross into the dark side of the apparatus, total time spent in the dark part of the box, and total crossovers between the light and dark sides were scored. Video was recorded via ANY-maze and hand-scored by an observer blinded to treatment status of the animals.

### Looming threat test

The looming threat test is modified from prior reports looking at the circuitry underlying unconditioned defense responses (28, 29). This test measures the species-typical reflex response to looming stimuli, i.e., freezing responses. In rodents, predators often strike from above, and an expanding visual stimulus thus triggers reflexive defense responses. Animals were placed into a transparent chamber (43.5 cm high × 18.5 cm diameter) with a computer screen placed above and a video camera placed beside to record changes in behavior. After a 2-min baseline period (solid gray screen, 23 lux) stimulus presentation was initiated. The stimulus consisted of a black dot which expanded from 2 to 20 degrees of visual angle over 250 ms. After reaching maximum size, the dot remained stable in size for 250 ms and then disappeared. This stimulus was repeated 15 times over a 22 s period with a 500 ms inter-stimulus interval. After the stimuli, the gray screen is presented until the experiment ends at 3 min; testing was conducted under 20 lux red light. Following behavioral testing, the videos were truncated into equivalent length periods (22 s each) and manually scored for freezing behavior by a blinded observer using the ANYMaze software. Freezing was defined as “ceasing” all activity, maintaining an attentive attitude at first, with head raised, eyes open, and body in the same position” (30).

### Acoustic startle response, startle habituation, and prepulse inhibition

The acoustic startle (ASR), startle habituation, and prepulse inhibition (PPI) protocols were adapted from our previous studies (31). All testing occurred within three sound attenuated startle chambers (SR-Lab Startle Reflex System; San Diego Instruments, San Diego, CA). The 15 min sessions consisted of a 5-min acclimation period with background noise (70 dB), 5 habituating startling stimuli (105 dB; 40 ms pulse), 2 blocks startling stimuli (93–123 dB, 40 ms pulse), 6 blocks of pseudorandom trials containing pulse-alone (105 dB; 40 ms) and prepulse-pulse (prepulses: 4, 8, and 12 dB above background noise; 20 ms), and 5 min post-test startling stimuli (105 dB; 40 ms pulse). During the prepulse-pulse trials, an inter-stimulus interval of 50 ms (onset to onset) was used. The inter-trial interval ranged from 15–30 s, randomly selected for each trial. Startle amplitude was defined as the peak piezoelectric accelerometer output over a 175 ms period beginning at the onset of the pulse stimulus.

### Sucrose preference test

The sucrose preference test used in this study was a modified version of the test previously described (15). Sucrose preference was measured over 5 days as followed: 4 consecutive days of 2 h exposure and 1 day of 2 h water restriction followed by 2 h exposure. Two bottles were available in each cage, one with 200 ml of 1% sucrose (w/v) and the other with 200 ml of tap water. At the end of the 2 h, the bottles were removed; consumption was noted, and the animals were returned to their previous housing conditions. Preference was measured as follows: total sucrose consumption (ml) / total sucrose consumption (ml) + total water consumption (ml).

### Novel object recognition test

The novel object recognition test (NORT) was a modified version of the test previously described by Bhardwaj et al. (32) and Ennaceur et al. (33). Novel object recognition test was performed in parallel in 4 enclosures (16′′ × 16′′ × 16′′, TruScan Arena, Coulbourn Instruments, Whitehall, PA). In order to standardize the salience of the objects, 2 options were printed on a commercial 3D printer (TAZ 6, Lulzbot): red cylinder (3.8 cm diameter, 3.5 cm height, untextured) and blue square (3.5 cm base length, 3.5 cm height, textured). Additionally, a set of gray objects were printed for test habituation: a half-egg (3.5 cm diameter, 3.5 cm height) and a pyramid (3.5 cm base length, 3.5 cm height).

The test consisted of habituation, training, and testing phases. For each test, objects were placed 20 cm apart, in the center of the cage. Rats were placed in the center of the box equidistant from both objects and preferences for objects were determined. The habituation and acquisition phases of the novel-object recognition test were each 5 min long, with a 5-min interval between the phases. The test phase was conducted 2 h after acquisition phase and also lasted 5 min. The three objects were randomly selected for each animal, and the cage placement of objects was randomized (left vs. right). After each run, objects and boxes were cleaned with 70% (v/v) ethanol to prevent odor cues. Automatic tracking of rats was performed with the detection of multiple body points (nose, middle, and tail) of the rat using the ANYMaze software. The time when the rat's nose was 2 cm from the object was defined as object exploration. The preference ratio (PR) for each animal was calculated from the time spent exploring the novel object (N) and the familiar object (F) during the test phase: PR = 100 × (N)/(N + F). Animals that failed to explore the objects for at least 10 s during the initial study phase were excluded from subsequent analyses (34).

### Electrode implantation

Twenty male animals (10 SD rats, 10 GEPR-3s) were implanted with a bipolar (twisted pair of stainless steel) electrodes (PlasticsOne) unilaterally targeting the DLSC 10–15 days after completion of all behavioral tests. SD rats and GEPR-3s were anesthetized with equithesin (a combination of sodium pentobarbital, chloral hydrate, magnesium sulfate, ethanol, and propylene glycol; 2.5 ml/kg, i.p.). Following anesthesia induction, animals were placed in a stereotaxic frame with animals positioned in the skull-flat plane. Electrodes were implanted in the DLSC using the coordinates (6.24 mm posterior to bregma, 1.0 mm lateral to the midline, and 3.7 mm ventral to the dura) from the atlas of Paxinos and Watson (35). Electrodes were fixed to the skull with three jeweler's screws using dental acrylic (Kooliner, GC America, Alsip, IL). Following recovery from anesthesia, rats were given caprofen (5 mg/kg, s.c.) as an analgesic and 1 ml warm normal saline (s.c.) to maintain hydration.

### Electrical stimulation of DLSC

One week after surgery, electrical stimulation of DLSC was conducted as described in Sahibzada et al. (36). Animals were placed into a circular acrylic chamber (30 cm diameter) and the implanted electrode was connected via a commutator (PlasticsOne) to a constant current stimulus isolation unit (AM Systems) triggered by a pulse generator (PulsePal). The testing session was comprised of a series of stimulations of ascending current amplitude, spaced a minimum of 15 s apart. The stimulation consisted of a 1 s train of negative monopolar square-wave pulses (0.2 ms) at a frequency of 100 Hz ranging in amplitude from 10 to 200 μA. After stimulation, the behavioral response was recorded, and the stimulating current was increased by 10 μA for the subsequent trial. A testing session was terminated either when the stimulating current reached 200 uA or when the animal displayed an escape behavior. Behaviors scored were binned into 3 categories: orienting, locomotion, and escape. Orienting was defined as a contralateral turning of the head, sometimes including turning of the body or circling behavior. Locomotion was defined as walking forward or “scooting,” a behavior that appeared as a walk with a small jump included. Escape behavior was defined as cringing or flinching movements, running, and jumping.

### Histology

Following the completion of all testing, rats were overdosed with deep equithesin (4 ml/kg) anesthesia and decapitated. Brains were fixed in 4% paraformaldehyde for a minimum of 72 h. After fixation, brains were cryoprotected in sucrose solution (30%) and frozen. Coronal brain sections (40 μm thick) were cut on a cryostat (Reichert Model 975C) and stained with cresyl violet acetate. Microscopic examination was performed to verify the location of electrode placement in DLSC according to the Swanson Brain Atlas (37). Electrode placement was performed blind to other data (behaviors evoked, stimulation thresholds).

### Statistics and data analysis

Statistical analyses were performed in GraphPad Prism (GraphPad Software, Inc., La Jolla, CA) and SPSS (Ver 25, IBM Corp). Open field, EPM, light-dark transition test, and sucrose preference test data were analyzed by two-way analysis of variance (ANOVA) with sex and strain as between-subject factors. PPI and startle amplitude data were analyzed by three-way ANOVA with sex and strain as between-subject factors and prepulse (or pulse) intensity as a repeated measure. Looming threat data were analyzed by three-way ANOVA with sex and strain as between-subject factors and test phase as a repeated measure. Startle habituation was analyzed by two-way ANOVA and by one-sample *t*-test against a test value of 1 (indicating no habituation). NORT data were analyzed by unpaired *t*-test (comparing SD rats and GEPR-3s) and by a one-sample *t*-test comparing performance to chance levels (test value 0.5). The proportion of animals that failed to explore objects during the test were analyzed by Fisher's Exact Test. Behaviors evoked by DLSC-stimulation were analyzed by two-way ANOVA with strain as a between-subject variable and behavioral category as a repeated measure. Correlations were assessed using Spearman's correlation on ranks, followed by the Benjamini-Krieger-Yukutieli's correction for false discovery rate (*Q* = 5%). Pairwise comparisons following all ANOVAs were analyzed using the Holm-Sidak correction for familywise error rate. *P*-values of < 0.05 were considered to be statistically significant.

## Results

### Open field test

**Figure 1:**
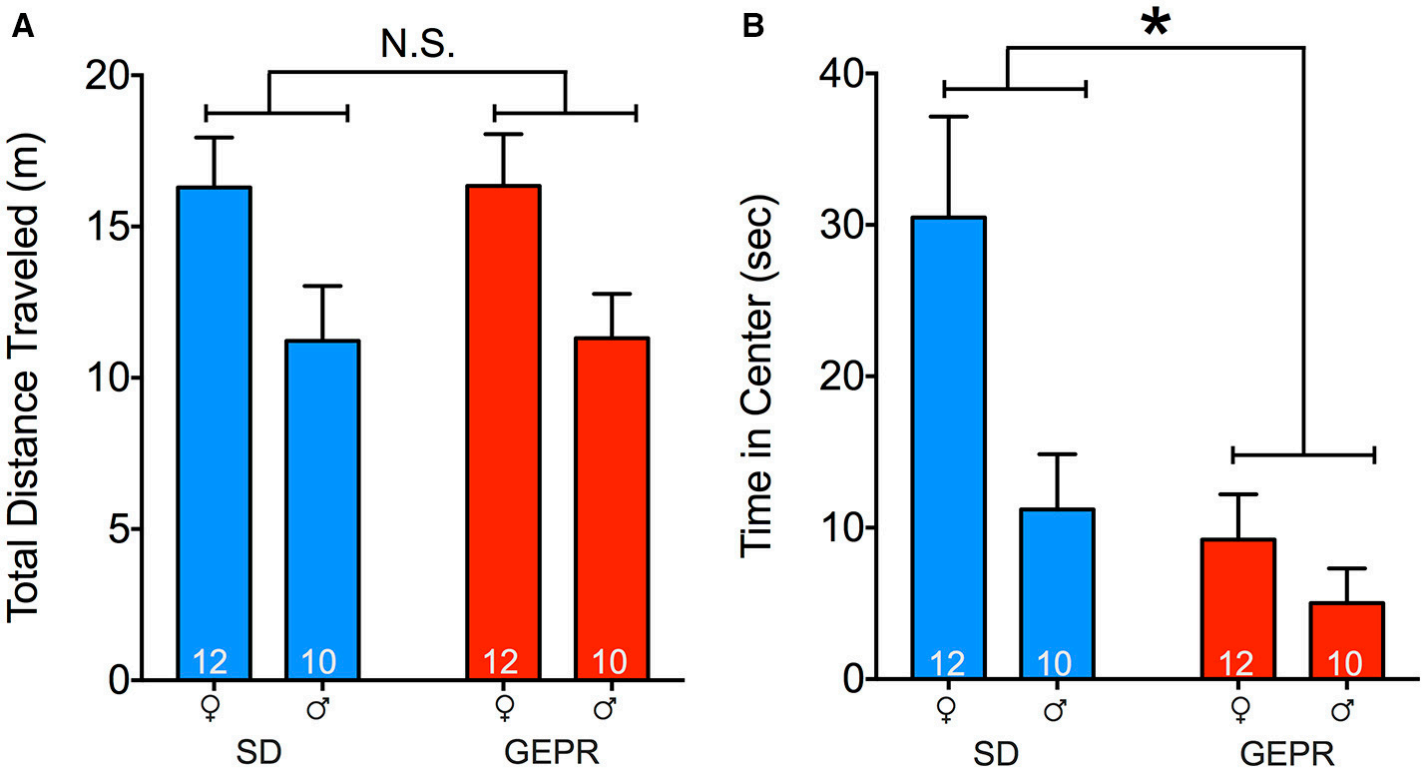
Open field test. **(A)** Total distance traveled (meters) for the duration of the test (10 min); males of both strains explored the arena less than females [*F*_(1, 39)_ = 9.0, *P* = 0.005; Holm-Sidak post-tests, *Ps* < 0.05]. **(B)** Total time spent exploring the center (8.5″ × 8.5″) of the open field. GEPR-3s explored less than SD rats [^*^F_(1, 39)_ = 9.2, *P* = 0.004], and males explored less than females [*F*_(1, 39)_ = 6.7, *P* = 0.01]. Post-tests also showed a decrease in exploration of the center of the arena in female GEPR-3s relative to SD rats (Holm-Sidak corrected, *P* < 0.05). Figures show mean and standard error of the mean.

Total locomotor activity in the open field did not differ as a function of strain (no main effect of strain, *P* = 0.97; Figure 1A). However, in both SD rat and GEPR-3 strains, males explored the arena to a lesser degree than did females [*F*_(1, 39)_ = 9.0, *P* = 0.005; Figure 1A Holm-Sidak post-tests, *Ps* < 0.05]. As a measure of anxiety-like behavior, we also measured the time spent exploring the center of the open field. There was a significant main effect of strain [*F*_(1, 39)_ = 9.2, *P* = 0.004; Figure 1B], a significant main effect of sex [*F*_(1, 39)_ = 6.7, *P* = 0.01; Figure 1B], but no sex-by-strain interaction (*P* = 0.1). Post-tests revealed a significant decrease in exploration of the center of the arena in female GEPR-3s as compared to female SD rats (Holm-Sidak corrected, *P* < 0.05). This did not reach the level of significance for males, likely due to a floor effect, as male SD rats explored the center of the arena for only a third of the time of females.

### Elevated plus maze

**Figure 2:**
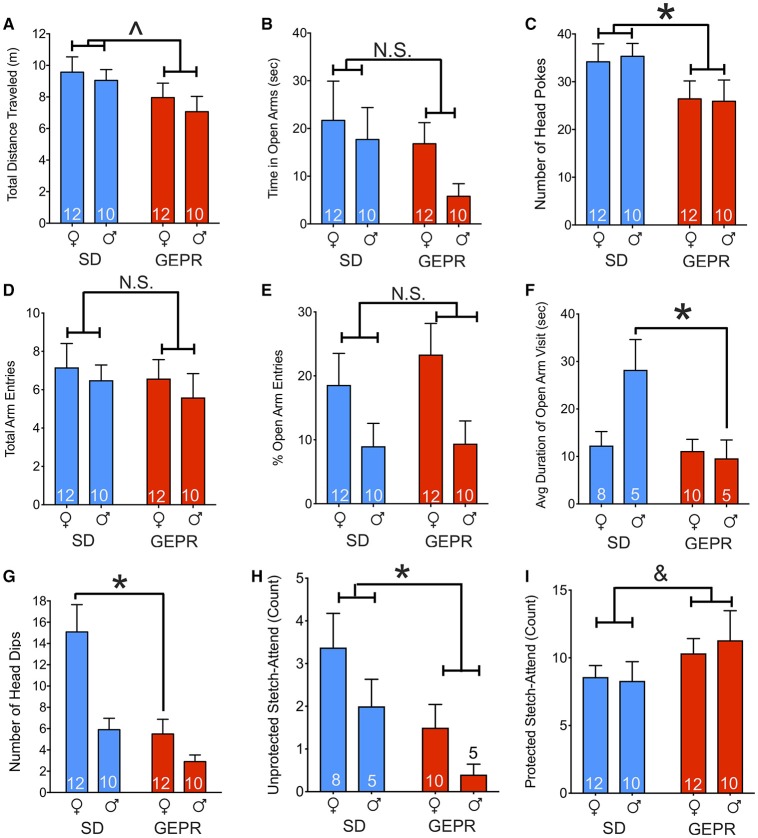
Elevated plus maze. **(A)** Total distance traveled in the maze for the duration of the test (300 s); GEPR-3s trending toward traveling less [∧*F*_(1, 40)_ = 3.9, *P* = 0.055]. **(B)** Time spent in the open arms of the EPM did not differ by either strain or sex (*Ps* = 0.18 and 0.23, respectively). **(C)** GEPR-3s displayed a decrease in number of head pokes into the open arms [^*^F_(1, 40)_ = 5.2, *P* = 0.03], but there was not an effect of sex. **(D)**, Total arm entries throughout the duration of the test did not differ significantly by either strain or sex (*Ps* = 0.50 and 0.46, respectively). **(E)** Percent open arm entries differed by sex [*F*_(1, 40)_ = 7.0, *P* = 0.01] but showed no effects of strain or strain-by-sex interaction (*Ps* = 0.56 and 0.63, respectively). **(F)** Average duration of open arm visit differed by strain [*F*_(1, 24)_ = 7.04, *P* = 0.01] and showed a significant interaction of sex-by-strain [*F*_(1, 24)_ = 5.499, *P* = 0.03], driven by males (^*^P = 0.009). **(G)** Number of head dips off the open arms differed by sex [*F*_(1, 40)_ = 7.04, *P* = 0.01] and strain [*F*_(1, 40)_ = 13.7, *P* = 0.0006]. The strain effect driven by females (*P* = 0.003). Accordingly, female GEPR-3s displayed fewer head dips than did female SDs (^*^P = 0.008). **(H)** Number of stretch-attend posture counts observed while the animal was in the “unprotected” portion of the maze (i.e., the open arms). SD rats has a higher frequency of this behavior as compared to GEPR-3s [^*^F_(1, 24)_ = 6.5, *P* = 0.018]. There was a trend toward an effect of sex [*F*_(1, 24)_ = 3.3, *P* = 0.08] but no strain-by-sex interaction (*P* = 0.8). **(I)** Number of stretch-attend posture counts observed while the animal was in the “protected” portion of the maze (i.e., the closed arms). There was a trend toward an effect of strain [^&^*F*_(1, 40)_ = 2.9, *P* = 0.098] with GEPR-3s showing a greater number of this risk-assessing behavior than SD rats. There was neither an effect of sex, nor a strain by sex interaction (*Ps* = 0.81 and 0.67, respectively). Figures show mean and standard error of the mean.

In the EPM, we detected a borderline-significant main effect of strain on total maze exploration [*F*_(1, 40)_ = 3.9, *P* = 0.055; Figure 2A], but neither a main effect of sex, nor a strain-by-sex interaction (*P* > 0.4 and *P* > 0.8, respectively). Time spent in the open arms of the EPM did not differ by either strain or sex (*Ps* = 0.18 and 0.23, respectively; Figure 2B). As a second measure of anxiety-like behavior, we examined the number of head pokes into the open arms and found a main effect of strain [*F*_(1, 40)_ = 5.2, *P* = 0.03; Figure 2C] but neither a main effect of sex nor a strain-by-sex interaction (*Ps* = 0.9 and 0.8, respectively). Total arm entries did not differ by either strain or sex [*F*_(1, 40)_ = 0.6 and 0.5; *Ps* = 0.5, respectively; Figure 2D]. Open arm entries relative to total arm entries (% open arm entries; Figure 2E) differed by sex [*F*_(1, 40)_ = 7.0, *P* = 0.01] but showed no effects of strain or strain-by-sex interaction [*F*_(1, 40)_ = 0.3 and 0.2; *Ps* = 0.6, respectively]. Average duration of open arm visit differed by strain [*F*_(1, 24)_ = 7.04, *P* = 0.01] and showed a significant sex-by-strain [*F*_(1, 24)_ = 5.499, *P* = 0.03] interaction, driven by males (*P* = 0.009; Figure 2F). Finally, we assessed head dips off the open arms (Figure 1G). We found a main effect of sex [*F*_(1, 40)_ = 7.04, *P* = 0.01] and strain [*F*_(1, 40)_ = 13.7, *P* = 0.0006], and a borderline significant interaction [*F*_(1, 40)_ = 3.6, *P* = 0.06]. Strain differed significantly only in females (*P* = 0.003) but not males (*P* = 0.24), and sex differences were only evident in SD rats (*P* = 0.0009) but not GEPR-3s (*P* = 0.29). Accordingly, female GEPRs displayed fewer head dips than did female SD rats (*P* = 0.008), consistent with increased anxiety-like behavior. Finally, we examined stretch-attend postures, an ethological measure of risk-assessment. The number of stretch-attend posture counts were divided into “protected” (when the animal was in the closed arms) and “unprotected” (while the animal was in the open arms). More anxious animals would be expected to have a higher stretch-attend phenotype in the closed arms. Indeed, SD rats has a higher frequency of this behavior as compared to GEPR-3s [main effect of strain: *F*_(1, 24)_ = 6.5, *P* = 0.018; Figure 2H]. There was a trend toward an effect of sex [*F*_(1, 24)_ = 3.3, *P* = 0.08] but no strain-by-sex interaction (*P* = 0.8). An increased number of stretch-attend posture counts observed while the animal was in the “protected” portion of the maze (i.e., the closed arms) may heightened risk-assessment (38–40). There was a trend toward an effect of strain [*F*_(1, 40)_ = 2.9, *P* = 0.098; Figure 2I] with GEPR-3s exhibiting a greater number of protected stretch-attend posture frequency than SD rats. There was neither an effect of sex, nor a strain by sex interaction (*Ps* = 0.81 and 0.67, respectively).

### Light-dark transition test

**Figure 3:**
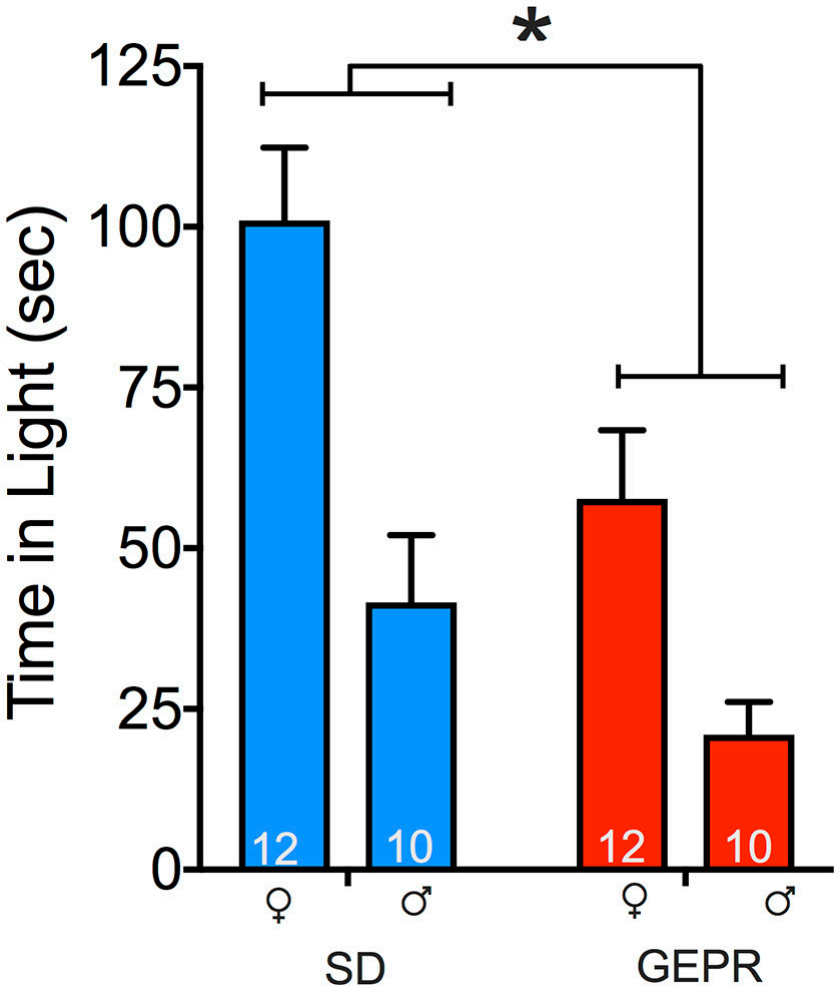
Light-dark transition test. GEPR-3s displayed reduced time spent in the light compartment [^*^F_(1, 40)_ = 10.1, *P* = 0.003] and males spend less time in the light than females [*F*_(1, 40)_ = 22.8, *P* < 0.0001]. Animals were initially placed in the light side of the apparatus; total test time was 5 min. Pairwise comparisons indicated a sex effect in both strains (*Ps* < 0.05, Holm-Sidak corrected), and the strain difference was significant for females, but not males (*P* < 0.05, Holm-Sidak corrected). Figures show mean and standard error of the mean.

Figure 3 shows time spent in the light compartment in the light-dark transition test. Consistent with the plus maze and open field, GEPR-3s displayed increased anxiety-like behavior in this test, as is evident from reduced time spent in the light compartment [main effect of strain: *F*_(1, 40)_ = 10.1, *P* = 0.003]. In addition to the main effect of strain, we found a main effect of sex [*F*_(1, 40)_ = 22.8, *P* < 0.0001], with male animals spending less time in the light than female animals. We did not find a strain-by-sex interaction (*P* = 0.3). Pairwise comparisons indicated that this sex difference reached the level of significance for both strains (*Ps* < 0.05, Holm-Sidak corrected), and that the strain difference reached the level of significance for females, but not male animals (*P* < 0.05, Holm-Sidak corrected). As with the open field, the lack of strain effect in male animals may be due to a floor effect.

### Looming threat test

**Figure 4:**
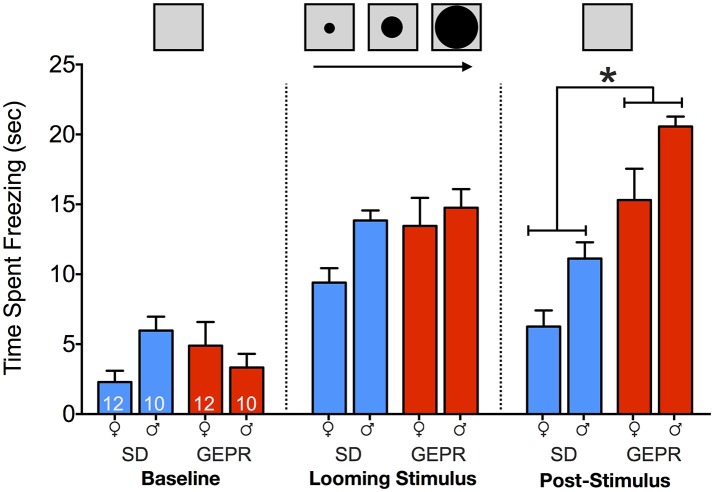
Looming threat test. Time spent freezing during: baseline period (22 s prior to stimulus presentation), presentation of looming stimulus (22 s), and in the post-stimulus period (22 s immediately after stimulus presentation). Overall, GEPR-3s spent more time frozen [*F*_(1, 40)_ = 11.7, *P* = 0.001], but there was no effect of sex (*P* = 0.12). During the post-stimulus period, there was a significant increase in freezing in GEPR-3s as compared to SD rats (^*^Holm-Sidak Adjusted, Females: *P* = 0.0004 and Males: *P* = 0.0007). Figures show mean and standard error of the mean.

In the looming threat test (Figure 4), we observed freezing as a measure of anxiety-like behavior during the: baseline period, presentation of looming stimulus, and in the post-stimulus period. We found a main effect of test period [*F*_(1.6, 63.8)_ = 113.1, *P* = 1 × 10^−19^], a main effect of strain [*F*_(1, 40)_ = 11.7, *P* = 0.001], but no main effect of sex (*P* = 0.12). In addition, we observed significant stage-by-sex [*F*_(1.6, 63.8)_ = 4.2, *P* = 0.03] and stage-by-strain [*F*_(1.6, 63.8)_ = 24.2, *P* = 0.00000003] interactions, but no other significant two or three-way interactions (*Ps* > 0.1). Pairwise comparisons revealed no strain differences during the baseline and stimulus presentation period, but a significant increase in freezing in GEPR-3s as compared to SD rats during the post-stimulus period (Holm-Sidak Adjusted, Females: *P* = 0.0004 and Males: *P* = 0.0007).

### Acoustic startle response, startle habituation, and prepulse inhibition

**Figure 5:**
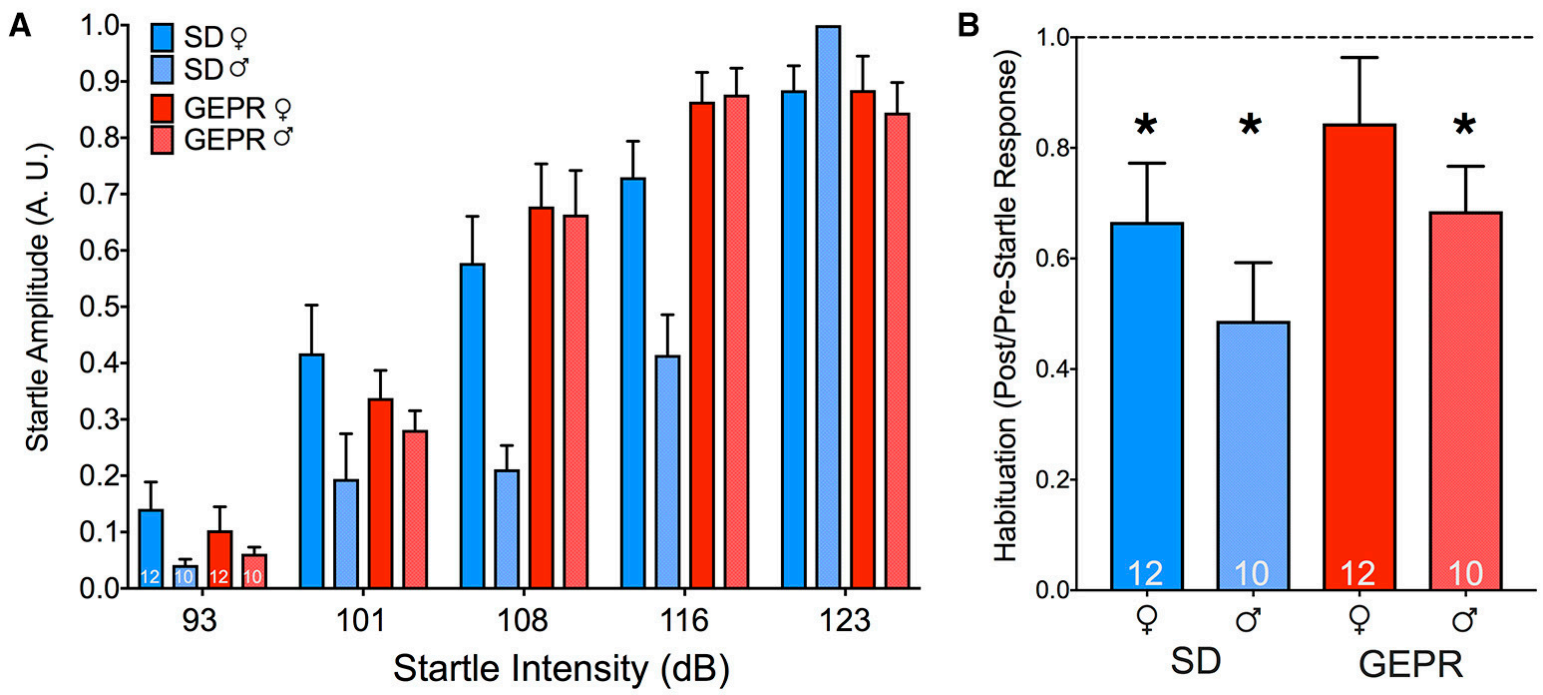
Acoustic startle response and habituation. **(A)** Startle amplitude (A.U.) as a result of increasing noise burst intensity (dB). We found a main effect of noise intensity [*F*_(1.6, 64.6)_ = 23.4, *P* = 0.0000001], but no effect of either strain or sex (*Ps* = 0.5 and 0.9, respectively). **(B)** Habituation to the startling stimulus was present in all groups, except for the female GEPR-3s (^*^P < 0.01, one sample *t*-test when compared to the theoretical mean of 1.0). Figures show mean and standard error of the mean.

As shown in Figure 5A, with increasing intensity of white-noise pulse, startle amplitude increased. The amplitude of the startle response was normalized to the maximum startle response within each subject to control for chamber-to-chamber variability in startle amplitude. We found a main effect of pulse intensity [*F*_(1.6, 64.6)_ = 23.4, *P* = 0.0000001], but no effect of either strain or sex (*Ps* = 0.5 and 0.9, respectively), nor any two- or three-way interactions (*Ps* > 0.2). As a second measure, we examined habituation of startle within a session (Figure 5B). We found that all groups showed the normal profile of habituation to the startling stimulus, except for the female GEPR-3s (*P* < 0.01, one sample *t*-test when compared to theoretical mean of 1.0). The main effect of strain approached but did not reach statistical significance [*F*_(1, 40)_ = 3.2, *P* = 0.085]; there was not a main effect of sex [*F*_(1, 40)_ = 2.513, *P* = 0.1208], nor an interaction [*F*_(1, 40)_ = 0.0089, *P* = 0.9]. The magnitude of startle response did not differ as a function of either sex or strain [Sex: *F*_(1, 40)_ = 0.05, *P* = 0.9, Strain: *F*_(1, 40)_ = 0.4, *P* = 0.5].

**Figure 6:**
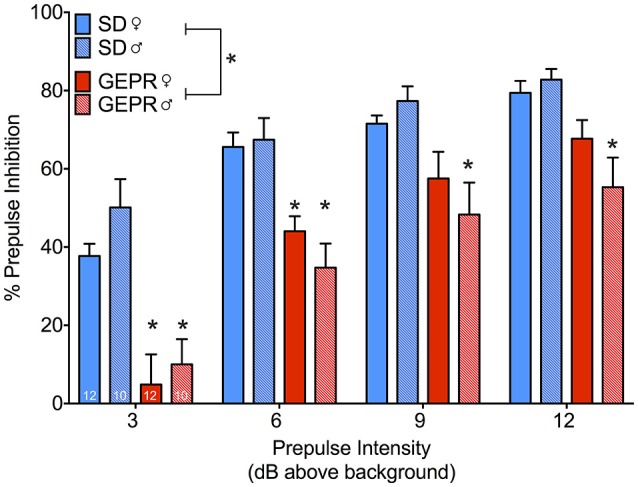
Prepulse inhibition of the acoustic startle response. All groups showed the expected increase in inhibition as a function of increasing prepulse intensity [*F*_(2.3, 93.1)_ = 82.2, *P* = 5 × 10^−23^], however GEPR-3s displayed a significant PPI deficit relative to SD rats [*F*_(1, 40)_ = 43.5, *P* = 0.00000007]. Comparisons across strain for each sex revealed significant impairment in PPI at each prepulse intensity for male GEPR-3s as compared to male SD rats (*Ps* < 0.002, Holm-Sidak adjusted). For female GEPR-3s as compared to female SD rats, this effect was evident at lower prepulse intensities (PP3: *P* = 0.005; PP6 *P* = 0.02), but not higher prepulse intensities (PP9 and PP12 *Ps* = 0.1). Figures show mean and standard error of the mean. ^*^significantl difference between strain, stratified by sex and prepulse.

We next assessed PPI (Figure 6), which measures a decrease in whole-body startle response when a startling stimulus is preceded by a low-intensity noise pulse. Analysis of PPI revealed the expected main effect of prepulse intensity [*F*_(2.3, 93.1)_ = 82.2, *P* = 5 × 10^−23^], as well as a main effect of strain [*F*_(1, 40)_ = 43.5, *P* = 0.00000007]. However, we found neither a main effect of sex, nor any significant two- or three-way interactions (*Ps* > 0.08). Collapsed across prepulse intensity, there were significant differences between the strains within each sex [Female: *F*_(1, 40)_ = 13.0, *P* = 0.001, Male: *F*_(1, 40)_ = 31.7, *P* = 0.000002]. Pairwise comparisons across strain for each sex revealed significant impairment in PPI at each prepulse intensity for male GEPR-3s as compared to male SD rats (*Ps* < 0.002, Holm-Sidak adjusted). For female GEPR-3s as compared to female SD rats, this effect was evident at lower prepulse intensities (PP3: *P* = 0.005; PP6 *P* = 0.02), but not higher prepulse intensities (PP9 and PP12 *Ps* = 0.1).

### Sucrose preference test

**Figure 7:**
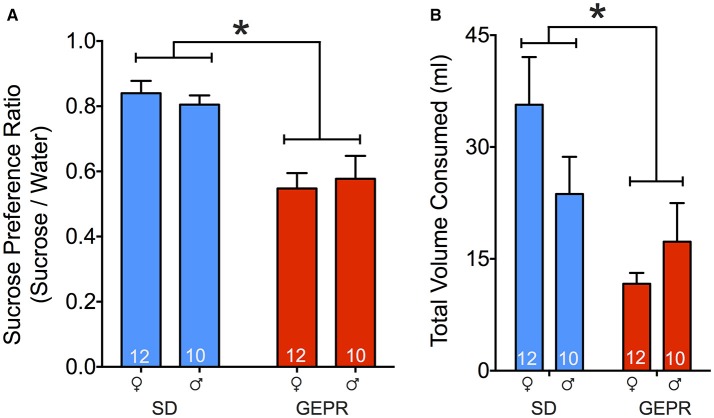
Sucrose preference test. **(A)** GEPR-3s displayed a significantly lower sucrose preference ratio than SD rats [^*^F_(1, 40)_ = 29.3, *P* < 0.0001]. Pairwise comparisons showed that decreased sucrose preference in GEPR-3s was significant in both sexes female: *P* = 0.001, male: *P* = 0.003, Holm-Sidak corrected. **(B)** Total volume consumed across days differed between strains [*F*_(1, 40)_ = 9.768, *P* = 0.0033], with GEPR-3s consuming significantly less than SD rats. Figures show mean and standard error of the mean.

To determine if comorbidities in the GEPR-3s extend beyond anxiety-like behavior and into symptoms related to depression, we next assessed hedonic response in the sucrose preference test. We calculated a sucrose preference ratio (vol sucrose consumed/vol water consumed) with total volume cumulated over the 5-day period of testing. We found a main effect of sex, with GEPR-3s displaying a significantly lower sucrose preference than SD rats [*F*_(1, 40)_ = 29.3, *P* < 0.0001; Figure 7A], but neither a main effect of sex, nor a sex-by-strain interaction (*Ps* = 0.96 and 0.5, respectively). Pairwise comparisons revealed that the decreased sucrose preference in GEPR-3s was significant in both sexes (female: *P* = 0.001, male: *P* = 0.003, Holm-Sidak corrected). Total volume consumed across days differed between strains [*F*_(1, 40)_ = 9.768, *P* = 0.0033; Figure 7B], with GEPR-3s consuming significantly less than SD rats, but not by sex [*F*_(1, 40)_ = 0.4239, *P* = 0.5187; Figure 7B].

### Novel object recognition test

**Figure 8:**
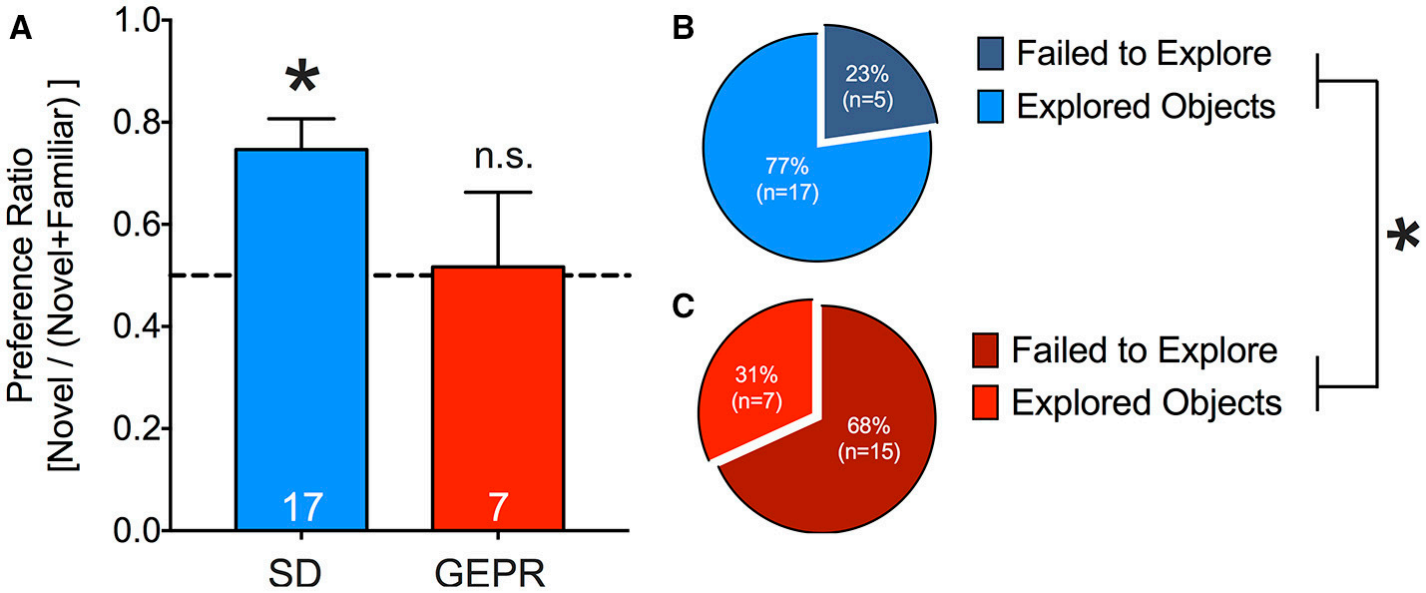
Novel object recognition test. **(A)** SD rats showed the expected novel object preference during the retention probe conducted 2 h after the familiarization session (one sample *t*-test, *t* = 4.1, *df* = 17, ^*^P = 0.0008), GEPR-3s did not (preference ratio did not differ significantly from chance, *P* = 0.9). Preference ratio trended toward but did not reach the level of statistical significance between these two groups (*t* = 1.7, *df* = 23, *P* = 0.09). **(B,C)** Proportion of animals that failed to explore the objects during the familiarization session; A greater proportion of GEPR-3s failed to explore the objects as compared to SD rats (^*^P = 0.006, Fisher's Exact Test). Figures show mean and standard error of the mean.

Because cognitive impairment has also been reported as a comorbidity of epilepsy, we next examined the performance of GEPR-3s as compared to SD rats in the NORT (Figure 8A). To our surprise, only a small proportion of GEPR-3s explored the objects, perhaps due to high levels of anxiety (Figures 8B,C). The proportion of animals that failed to explore the objects was significantly greater in the GEPR-3 strain as compared to the SD rat strain (*P* = 0.006, Fisher's Exact Test). Because of this attrition, we collapsed across sex for the recognition memory trial (Figure 8A). While SD rats showed the expected preference for the novel objects (one sample *t* test, *t* = 4.1, *df* = 17, *P* = 0.0008), GEPR-3s did not (preference ratio did not differ significantly from chance, *P* = 0.9). Preference ratio trended toward but did not reach the level of statistical significance between these two groups, likely due to the low statistical power due to attrition in the GEPR group (*t* = 1.7, *df* = 23, *P* = 0.09).

### Electrical stimulation of DLSC

**Figure 9:**
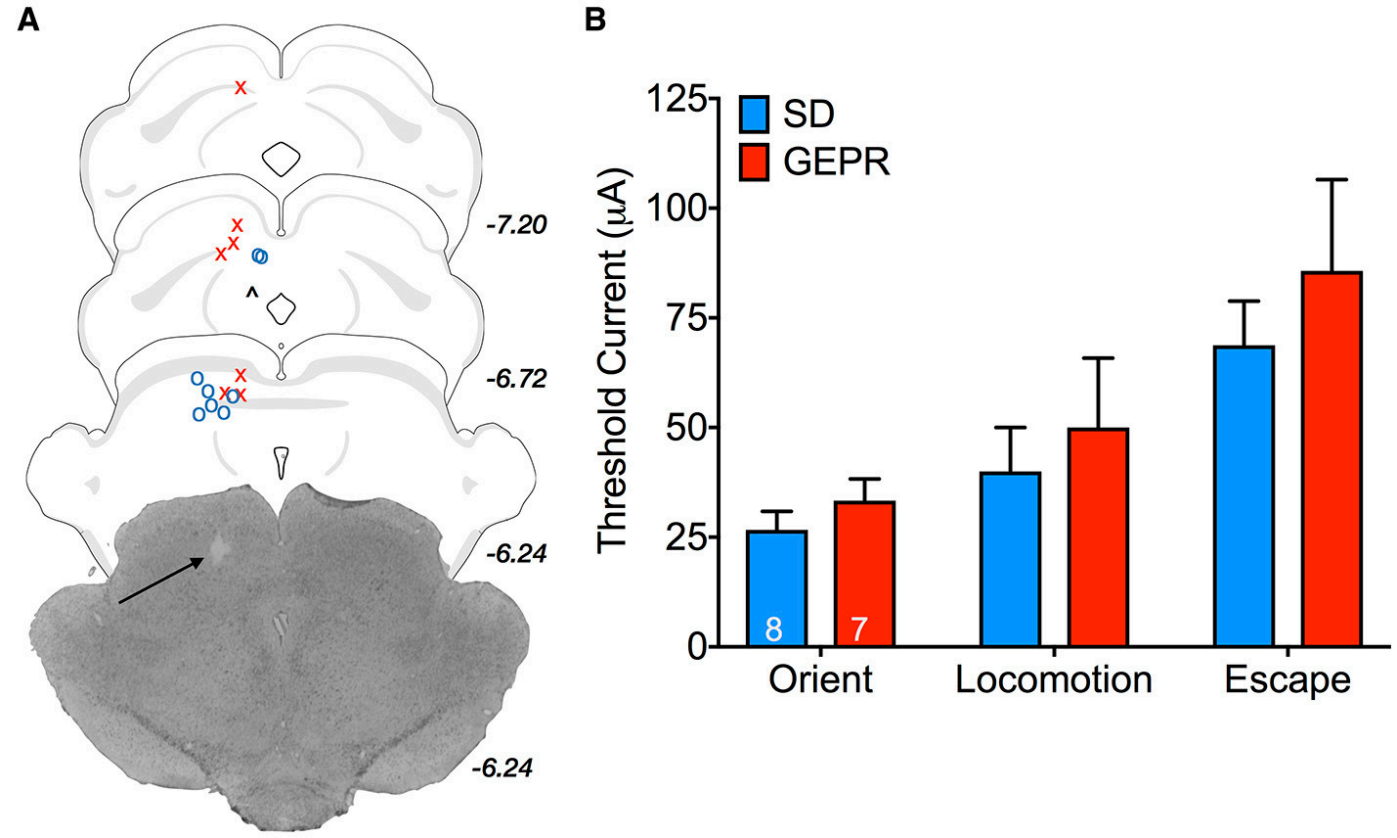
Stimulation thresholds for DLSC-evoked behaviors in male rats. **(A)** Electrode placement; O = SD rats, X = GEPR-3s, ∧ = misplaced electrode. Black arrow points to electrode tip in the representative photomicrograph. Electrode tips are plotted on planes from the BrainMaps 4.0 Atlas (37). **(B)** Threshold current required to evoke orienting, locomotor responses, or escape behaviors. No differences between strain (Orient: *t* = 1.026, *df* = 10, *P* = 0.33 Locomotion: *t* = 0.488, *df* = 5, *P* = 0.65 Escape: *t* = 0.765, *df* = 13, *P* = 0.46). Figures show mean and standard error of the mean.

Activation of components of the midbrain tectum result in species-specific defense responses (41–44). With increasing stimulation intensities, progressively more severe responses are evoked. For these studies, only male animals were available for use. Two SD rats and 2 GEPR-3s were excluded from analysis due to either: loss of electrode-containing head cap, lack of response to DLSC stimulation, postsurgical mortality, or inability to verify electrode placement. Electrode placement across groups is shown in Figure 9A; one GEPR-3 was excluded due to misplacement of the electrode in the superficial SC or the PAG (black “∧” in Figure 9A). A representative photomicrograph of electrode placement is shown in Figure 9A. When we measured the threshold current required to evoke orienting, locomotor responses, or escape behaviors, we found no differences between strain (Figure 9B; Orient: *t* = 1.026, *df* = 10, *P* = 0.33 Locomotion: *t* = 0.488, *df* = 5, *P* = 0.65 Escape: *t* = 0.765, *df* = 13, *P* = 0.46).

### Correlation of behaviors

To summarize our findings across tests, and to determine the degree to which behaviors may be considered “trait-anxious” (i.e., independent of the particular context), we correlated dependent measures across the tests in which we detected an effect of strain (45, 46). Table 1 shows the Spearman's correlation coefficients for these comparisons. Correlations indicated in bold were considered discoveries using the Benjamini-Krieger-Yukutieli correction for false discovery rate (*Q* = 5%). Of the measures we examined, the post-stimulus freezing time in the looming threat test was best correlated with other measures.

**Table 1:** Spearman's correlation coefficients for characterizing behaviors consistent with “trait anxiety.”

	**EPM: head pokes**	**Looming threat: post-stimulus freeze time**	**Light/dark: light time**	**OF: inner time**	**Sucrose pref ratio**	**PPI: average**
EPM: head pokes						
Loomer: post-stimulus freeze time	−0.288					
Light/Dark: light time	0.189	−**0.483**				
OF: inner time	0.150	−**0.482**	0.210			
Sucrose pref ratio	0.130	−**0.535**	**0.426**	0.175		
PPI: average	0.259	−**0.375**	0.127	**0.348**	**0.336**	

*Correlations indicated in bold were considered discoveries using the Benjamini-Krieger-Yukutieli correction for false discovery rate (Q = 5%). The post-stimulus freezing time in the looming threat test was best correlated with other measures*.

## Discussion

Here, we report that the GEPR-3 strain exhibits anxiety-like behaviors in both sexes and across an array of standard behavioral assays (open field, EPM, and light-dark transition test). Moreover, when tested in a novel implementation of a looming threat test, GEPR-3s demonstrated heightened anxiety-like responses. In addition, GEPR-3s displayed disrupted PPI of the acoustic startle response in the absence of changes in startle reactivity, reduced preference for sucrose, and impaired novel object recognition.

Altered behavioral responses in tasks thought to reflect affective state have been previously reported in animal models of epilepsy, although results have varied. For instance, following status epilepticus, both *increased* and *decreased* anxiety- and depression-like behaviors have been reported (47–50). Electrical and chemical kindling epileptogenesis are also associated with affective comorbidities including increased defensive, anxious-, or depressive-like phenotypes (51–56). However, similar to status epilepticus models, some kindling epileptogenesis studies have failed to find effects on comorbidities (57–59). Anxious- and depressive-like comorbidities have also been reported in GAERS (60); however, these comorbidities differ between sub-colonies of the strain (61), and are evident only when GAERS are compared to non-epileptic, inbred control rats but not when compared to the outbred strain from which they were derived, the Wistar rat (62). WAG/Rij rats, which also display absence-like seizures, have a co-morbid depressive-like phenotype, but not an anxiety-like phenotype (7, 63, 64).

While the above studies address the effects of acute seizures and/or a history of recurrent seizures on comorbidities, AGS models of inherited epilepsy allow for the assessment of phenotypes, i.e., those associated with underlying pathology or genetics in the absence of recurrent seizure activity. AGS models such as the GEPR, the Wistar audiogenic rat (WAR), and Kurshinsky-Molodkina (KM) rat display increased susceptibility to acoustically-evoked generalized seizures, which are common in models of inherited epilepsy across species (64–66). Of these strains, the WAR and KM strains have been evaluated for comorbidities (67, 68). While there has been some suggestion that the GEPR-3 strain may also display affective comorbidities (69, 70), our data clearly demonstrate that comorbidity in GEPR-3s is an anxiety-like phenotype. Across standard tests of conflict-exploratory activity, GEPR-3s consistently displayed reduced exploration of aversive maze components. In the open field test, this was manifest as a reduced exploration of the center of the arena, in contrast with the increased exploration reported in WARs (67). In the EPM test, anxiety-like behavior was evident in fewer exploratory headpokes into the open arms of the maze, which is consistent with reports in KM rats (68). In the light-dark test, GEPR-3s had reduced time spent in the light chamber of the apparatus; this phenotype extends beyond conflict-exploratory tests into unconditioned fear: when challenged with a looming visual stimulus, GEPR-3s displayed increased freezing in the post-stimulus period.

The anxiety-like phenotype observed in GEPR-3s may be in part explained by neurochemical abnormalities; these animals display deficits in brainstem serotonin, which is a well-known regulator of affective function (71–74). Consistent with the reduced serotonin levels reported in GEPR-3s, we have previously reported volumetric alterations in the region of the dorsal raphe nucleus (11). Serotonin is also a regulator of seizure susceptibility in GEPR-3s (69, 75), and accordingly, treatment with fluoxetine, a selective serotonin reuptake inhibitor that is primarily used for the treatment of depression and generalized anxiety, results in a decrease in AGS severity (9). In KM rats, fluoxetine, reduced immobility in the forced swim task (68), although it had no effect on EPM behavior. The degree to which serotonin-based pharmacotherapy would normalize behavioral co-morbidities in the GEPR-3 remains to be explored.

Anxiety-like responses were also observed in the NORT, where a large proportion of GEPR-3s failed to explore novel objects. This phenotype was at least as striking as the memory impairment evident in the subset of GEPR-3s that did explore objects. While a subset of GEPR-3s explored the objects to an extent sufficient to perform the test, the impairment seen in object recognition memory should be interpreted with caution because the anxiety phenotype may have impaired memory consolidation in GEPR-3s. While the mechanism(s) underlying impaired learning/memory in GEPR-3s is unknown, it is worth noting that heightened levels of corticosterone have been associated with impaired learning/memory, including NORT performance (76). Interestingly, GEPR-3s and WARs have elevated corticosterone levels (82, 83). These same caveats must be considered when interpreting the decreased sucrose preference in GEPR-3s. While these data are consistent with a decreased hedonic drive, they may also have resulted from anxiety-induced suppression of feeding (84). In fact, a recent study shows that administration of anxiolytic drug fluoxetine results in a recovery of feeding behavior in a corticosterone-induced rodent model of anxiety (85). Future studies of GEPR-3s investigating changes in reward and learning behaviors under conditions of similarly reduced anxiety and/or in the presence of anxiolytic drugs may help to parse these effects.

The DLSC play a key role in the generation of the wild running component of AGS in the GEPR (86). Both functional and anatomical evidence suggests that the DLSC are abnormal in the GEPR; GEPR-3s display an increase in DLSC volume as compared to SD rats (11), and neurons within the DLSC of the GEPR-9 (a substrain of the GEPR that exhibit tonic seizures) display reduced sensitivity to acoustic stimulation relative to SD rats (86). On the basis of these findings, we hypothesized that GEPR-3s would display altered thresholds for DLSC-evoked behavioral responses; however, stimulation thresholds did not differ between strains. This was also surprising given the pronounced increase in freezing seen in the looming threat task, which critically relies upon the DLSC (29, 43, 87, 88). However, the DLSC is only one component of a midbrain network mediating both defensive responses and AGS; other loci include the inferior and superior colliculi, and periaqueductal gray (36, 89–92). Other nodes may be relevant for epilepsy-associated anxiety responses; for example, electrical kindling of the amygdala coincident with PAG stimulation exacerbates panic-like behaviors evoked from the PAG (55). Evaluation of thresholds for defense responses from these other sites may be merited in the GEPR.

The midbrain network mediating both defensive and AGS also play an important role in the control of acoustic startle and PPI. Lesions to either the inferior colliculus (IC) or superior colliculus (SC) disrupt PPI, whereas electrical or chemical stimulation of the IC or SC increases PPI (77–81). Importantly, the duration of tone burst intensity required to elicit AGS (10–60 s) is far longer than the noise burst used to induce auditory startle (40 ms pulse) (14), thus it seems unlikely that seizure activity could account for the deficits in PPI seen in GEPR-3s. The PPI deficits in GEPR-3s may be related to the same underlying pathology as the anxiety-like behaviors as serotonin modulates PPI in rodents (93, 94), and human studies report disrupted PPI in patients with panic disorder (95, 96). However, while kindling epileptogenesis disrupts PPI (97, 98), GAERs display either normal or facilitated PPI depending on the age tested (99), suggesting that PPI deficits are not a universal comorbidity of seizures in animal models.

There were some notable cases in which GEPR-3s did not display heightened anxiety responses: open arm exploration in the EPM, acoustic startle, and electrical stimulation of the DLSC. Open arm exploration in the EPM is impacted by a variety of variables, including prior test experience (100) and history of handling (101). Prior exposure to novel environments may reduce subsequent exploratory drive in the EPM, diminishing ability to distinguish anxiety-like responses. However, it is worth noting that despite the lack of difference in open arm exploration, other measures of ethological activity in the EPM revealed a consistent anxiety-like phenotype in the EPM. Acoustic startle and electrical stimulation both differ from the conflict-based tasks (exploration vs. safety): these are unconditioned or evoked responses. In the cases in which anxiety-like responses were detected, animals were typically presented with a novel environment (or object) to explore, suggesting that at least part of the anxiety phenotype in GEPR-3s may be related to neophobia, and does not generalize to anxiety-like responses that may be more related to panic or acute fear.

There is an abundance of clinical evidence supporting sex differences in acquisition and expression of seizure disorders (102–104). Proposed etiologies of these sex-based differences include developmental mutations (as in the case of protocadherin 19 mutations, an X-linked gene), neuroendocrine fluctuations (e.g., perimenstrual catamenial epilepsy), and changes in neuroinflammatory response (103, 105). Some of these same mechanisms underlying sex differences in epilepsy may also contribute to divergent rates of comorbidities in males and females (13). Although women in the general population are more likely to show signs of anxiety and depression, sex appears to have a protective effect in patients with epilepsy—as they age, men become more susceptible to depression and women become less susceptible (106). The penetrance of the GEPR-3 seizure's phenotype is significantly greater in females as compared to males (107), although in the present study we found no notable sex-by-genotype interactions: anxiety-like responses were equally present in both female and male GEPR-3s. However, the effect of repeated seizures on these comorbidities remains unknown—future studies examining effects of repeated seizures on behavioral phenotype and potential influences of sex are clearly needed.

Here we demonstrate for the first time an anxiety phenotype in adult GEPR-3s; this phenotype was present in animals that experienced only a single AGS at PND 21. In the GEPR-3, anxiety-like responses were evident across a variety of tasks and conditions. Given the minimal seizure history, neuropsychiatric phenotypes in the GEPR-3 are likely *premorbid* rather than *comorbid*; this feature strongly suggests a genetic component of etiology of anxiety that provides a novel approach for future investigations of the pathogenesis of anxiety in epilepsy.

## Author contributions

BA and PF: designed experiment; BA: conducted experiment; BA and PF: analyzed data; BA, LM, PN, and PF: interpreted findings; BA and PF: drafted manuscript; BA, LM, PN, and PF: edited and approved manuscript.

### Conflict of interest statement

The authors declare that the research was conducted in the absence of any commercial or financial relationships that could be construed as a potential conflict of interest.
